# Impact of Telehealth Care among Adults Living with Type 2 Diabetes in Primary Care: A Systematic Review and Meta-Analysis of Randomised Controlled Trials

**DOI:** 10.3390/ijerph182212171

**Published:** 2021-11-19

**Authors:** Natalie Robson, Hassan Hosseinzadeh

**Affiliations:** School of Health and Society, University of Wollongong, Wollongong, NSW 2525, Australia; hassanh@uow.edu.au

**Keywords:** type 2 diabetes, diabetes mellitus, telehealth, telemedicine, telecare

## Abstract

Primary health care physicians are increasingly offering telehealth services to patients not only for its cost and time saving advantages but for the additional benefits telehealth can provide for patients with type 2 diabetes (T2D) such as improved self-management behaviours. To support the development of telehealth based T2D clinical care models in primary health care settings, a narrative synthesis and meta-analysis of randomised controlled trial studies was completed for 29 studies that evaluated the effect of one or more types of telehealth interventions on HbA1c levels compared to usual care alone. Results from the random effects meta-analysis demonstrated that telehealth interventions had a stronger influence on HbA1c compared to usual care with a mean difference in HbA1c \% −0.18 (CI −0.35, −0.01), *p* = 0.04. Results from the subgroup meta-analysis demonstrated that telehealth interventions, when grouped by type of telemonitoring (mHealth and telephone communication), all have a stronger effect on lowering HbA1c levels; however, none of these findings were significant. Key findings from this review demonstrate that telehealth interventions that address T2D self-management behaviours and have higher levels of health care provider engagement, have greater effects on lowering HbA1c levels compared to usual care alone.

## 1. Introduction

The term “telehealth” is defined by the International Organisation for Standardisation as the ”use of telecommunication techniques for the purpose of providing telemedicine, medical education, and health education over a distance” [1]. Whilst the terms “telehealth” and “telemedicine” are often used interchangeably, it is important to recognise the subtle distinction between the two terms. The World Health Organisation defines telemedicine as “the delivery of health care services, where distance is a critical factor, by all health care professionals using information and communication technologies for the exchange of valid information for diagnosis, treatment and prevention of disease and injuries, research and evaluation, and for the continuing education of health care providers, all in the interests of advancing the health of individuals and their communities” [2]. Whilst these descriptions are quite similar, the term “telehealth” encompasses all forms of remote health care services whereas “telemedicine” describes the remote delivery of “clinical” services [2]. A key example of a non-clinical telehealth health service might involve a telephone call to provide healthy lifestyle coaching or generalised health advice. 

Primary care physicians are increasingly offering remote care services to their patients through video, telephone and web/mobile-based applications as they recognise the benefits that telehealth can bring such as health cost savings, greater patient compliance with treatment plans [3], improved communication between health care providers and patients [4,5,6,7] and early identification of abnormalities [8]. 

Telehealth also provides a unique opportunity for primary health care professionals to reach socially disadvantaged groups where cost, geographic location or cultural barriers may limit health service access [2,9]. Whilst the use of technology may not be as easily accessible for older, ethnically diverse or socially disadvantaged groups [10], the potential application of telehealth services to address health inequalities amongst vulnerable population groups should not be underestimated. 

In the wake of the recent global pandemic, primary care physicians had no choice but to embrace the use of technology to provide remote delivery of care to help reduce the spread of the SARS-COV-2 virus [11]. In many ways, the global pandemic provided an opportunity for the world to experience the remote delivery of primary health care services, something that had failed to gain traction in earlier years due to a lack of interest and funding [11]. 

Whilst there will always be a need for face-to-face care delivery in primary health care settings, people accessing face-to-face care for routine patient monitoring related to the management of chronic health conditions such as type 2 diabetes can benefit from telehealth services [12]. 

The global prevalence of diabetes is estimated to affect 463 million people, with type 2 diabetes representing around 90% of cases [13]. By the year 2030, it is expected that the global prevalence will continue to grow to 578 million [13]. Between the years 2000 and 2019, there was a 70% increase in the number of global diabetes related deaths which placed diabetes in the top 10 causes of death in the year 2020 [14]. Such figures highlight the present-day challenges primary health care services face in regard to type 2 diabetes management and care. 

Current best practice related to the management of people living with type 2 diabetes focuses on the prevention of diabetic related complications and disease progression through healthy diet and lifestyle related factors such as exercise and medication [15,16,17,18]. In the absence of acute diabetic related complications, many routine medical appointments with a general practitioner would involve healthy lifestyle education and medication prescription, both of which have been shown to be just as effective when delivered in remote settings [12,19,20,21]. An increasing number of randomized controlled trials have compared the impact of telehealth on diabetes management with usual care and reported conflicting findings. For instance, some studies found that telehealth leads to better blood glucose control [22,23] while other studies did not support this [24].

Whilst there are a number of systematic reviews that have evaluated the efficacy of telehealth interventions for the management of type 2 diabetes, there is limited information in the literature when it comes to the evaluation of telehealth interventions that serve as a substitute to traditional face-to-face consultation in primary health care settings. This study aims to fill this gap by comparing telehealth interventions to standard face-to-face care among people living with type 2 diabetes through a systematic review and meta-analysis. The findings of this study contribute to the growing body of evidence about the use of telehealth for type 2 diabetes management while providing greater specificity around the frequency, volume and type of interventions to inform best practice guidelines for primary health care clinicians. 

## 2. Methods

### 2.1. Search Strategy 

A detailed search of the literature was completed between 16/5/21–22/6/21 using the following search string: (([type 2 diabetes OR diabetes mellitus] AND [telehealth OR telemedicine]) AND (usual care OR standard care OR standard treatment)), AND (Randomised controlled trial OR randomized controlled trial or RCT). 

As the terms diabetes mellitus and type 2 diabetes can be used interchangeably, diabetes mellitus was included as a search term as it sought to capture as many type 2 diabetes studies as possible. Whilst diabetes mellitus also includes both type 1 and gestational diabetes, studies that recruited subjects who had type 1 or gestational diabetes were excluded from this review. The terms “telehealth” and “telemedicine” were used as overarching search terms to capture all forms of telehealth technologies in an attempt to reduce the number of duplicated studies obtained in the search. Limitations associated with the decision to not include specific telehealth technology search terms such as “mHealth”, “telemetry”, etc., are discussed in the limitations section of this review. 

Boolean operators ‘OR’ and ‘AND’ were used to connect similar searches to combine key search terms as defined in the search strategy. Studies were searched using the following search string across 5 key scientific databases PubMed, Scopus, Cochrane library, Web of Science and CINAHL with title and abstract screening completed as the first stage of data extraction. A set of inclusion and exclusion criteria were defined prior to the first stage of title and abstract screening to support study rigor and the specificity of search results. Inclusion criteria for the following search included studies that were full text, available in English, peer reviewed, published between January 2011 and September 2021 and with a study design utilising a randomised controlled trial design. A list of the study exclusion criteria can be found in the table below.
**Exclusion Criteria**Studies that focus on ‘telerehabilitation’ or ‘telepharmacy’Multimorbidity studies, subjects with type 1 or gestational diabetesStudies that do not meet RCT checklist criteriaSubjects < 18 years of agePilot studiesLow sample size < 50 participants in studyHbA1c not listed as a primary or secondary outcome measureGrey literature

**Figure 1:**
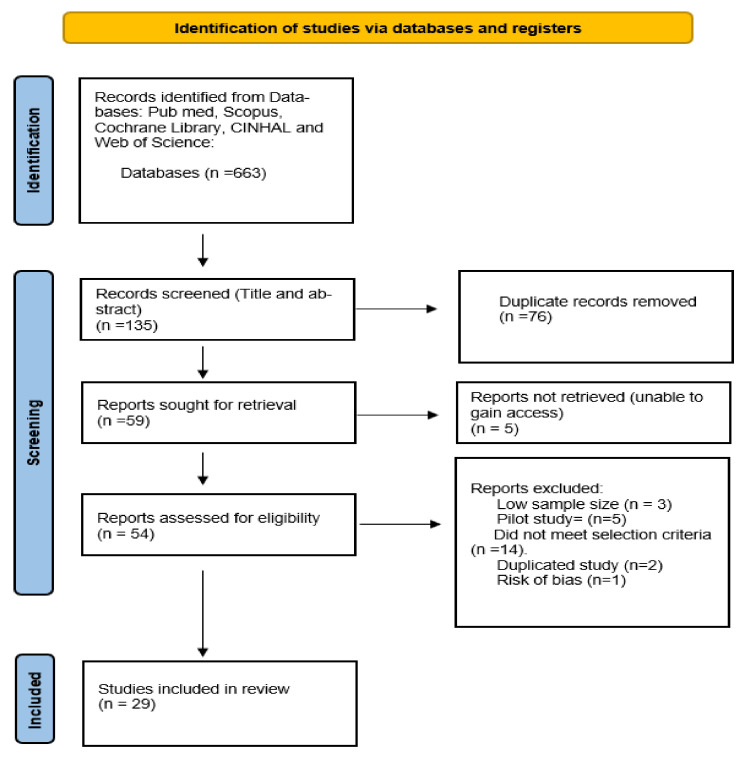
PRISMA flow diagram.

Of the 5 databases searched, a total of 663 citations were identified. After title and abstract screening, a total of 135 records were obtained and transferred to endnote referencing management software. Prior to the second stage of screening, 76 duplicate records were removed yielding a total of 54 records for further screening as 5 articles were unable to be retrieved. Of these studies, a further 25 studies were removed for reasons outlined in the PRISMA flow diagram (see Figure 1). A total of 29 studies were included in the systematic review. Two authors were involved in the selection process and any disagreement was solved using discussions.

### 2.2. Risk of Bias Assessment 

A risk of bias assessment was completed for all studies included in the systematic review using the Cochrane Back Review Group (CBRG) assessment criteria [25]. For studies to be considered as a part of the final narrative synthesis, they were required to meet 6 or more criteria (indicated by a yes response) using the CBRG assessment tool to indicate low study bias. Once the primary researcher had completed the risk of bias assessment on all selected articles, the process was then repeated by the lead reviewer who had agreed to remove one of the selected studies due to a high risk of bias score. The risk of bias assessment for all studies in this review can be found in the Appendix (see Appendix A). 

### 2.3. Systematic Review Analysis 

The first stage of data analysis involved data extraction from the final section of the RCT studies following completion of the risk of a formal bias assessment. Key data extracted from the studies included study characteristics, participant characteristics, primary and secondary outcome measures, study results and a brief description of the telehealth interventions (see Table 1). The data extraction process was completed manually using Microsoft Excel. Through the process of data extraction, we explored various themes surrounding telehealth interventions for type 2 diabetes management such as HbA1c control—the most commonly utilised telehealth technology for type 2 diabetes management—length of intervention, and the influence of potential confounding factors of socioeconomic status.

### 2.4. Meta-Analysis 

A quantitative meta-analysis was conducted as a part of this systematic review using RevMan (Review manager software version 5.4) (Cochrane, London, United Kingdom). RevMan is used for extracting study data, preparing the characteristics of studies and developing comparison tables for systematic reviews. It is also used for conducting meta-analysis and generating graphics for the results. Pooling together the results and conducting meta-analysis improves the precision of the estimation of the effect of interventions and settles conflicting findings from different studies. The purpose of the meta-analysis is to examine any differences between the intervention and control groups across all selected studies by pooling together the results of studies that provided a mean HbA1c% and standard deviation as either a primary or secondary outcome measure. Meta-analysis was also completed for body mass index (BMI), as a secondary outcome measure for BMI has been shown to influence type 2 diabetes related outcomes [26]. A total of 18 studies were selected for meta-analysis. The remaining studies had either missing or incomplete datasets so could not be considered for the meta-analysis. 

Once all of the relevant data was manually entered into RevMan, a random effects meta-analysis was conducted utilising inverse variance as the statistical method and mean difference as the effect measure with a 95% CI (confidence interval) to compare the combined effectiveness of telehealth interventions. The subgroup analyses were conducted for telemonitoring (telehealth studies that used telemonitoring technology like wearable devices for type 2 diabetes management), mHealth (telehealth studies that used interventions delivered via mobile technologies for type 2 diabetes management), telephone communication (telehealth studies that used the telephone for type 2 diabetes management) and self-management (telehealth studies that used self-management interventions for type 2 diabetes management) focused interventions. As a minimum of two studies were used to conduct a meta-analysis [27], it was not possible to perform subgroup analysis for virtual consultation and video education categories as there was only one study in each category that had available data [28,29]. This was also the case when it came to further exploring the relationship between socioeconomic status and telehealth efficacy. 

## 3. Results 

A total of 29 RCTs that evaluated the effectiveness of telehealth interventions on the management of type 2 diabetes were included in the narrative syntheses. As outlined in Table 1, the most commonly utilised telehealth intervention amongst the selected studies involved telemonitoring (17 studies), mHealth (10 studies), telephone communication (3 studies), virtual consultation (web) (2 studies) and video education (1 study). Five of the total studies in this review utilised a combination of telemonitoring and mHealth interventions [5,30,31,32,33], two studies utilised both telemonitoring and video conferencing [9,34] and one study utilised both telemonitoring and telephone coaching as a part of the intervention [35].

### 3.1. Length of Intervention

The duration of interventions ranged from 3 months to 5 years; however, the majority were 12 months (11 studies) and 6 months (10 studies) in duration. 

### 3.2. Age Range and Average Range of Participants

The average age of participants across studies was 56.5 years for all studies with an age range of 38.5 and 78.1 years.

### 3.3. Telemonitoring 

Of the 17 studies that had evaluated the effectiveness of telemonitoring for type 2 diabetes management, 9 studies [4,5,8,31,33,34,35,36,37] reported statistically significant changes between the telemonitoring intervention groups and control groups measured by a reduction in mean HbA1c levels. 

For the studies that failed to see statistically significant intergroup differences in mean HbA1c, some statistically significant improvements were seen in secondary outcome measures including a reduction in outpatient, Emergency Department (ED) visits as well as planned hospitalisations [38], and a significant reduction in LDL (low-density lipoprotein) cholesterol levels among the telemonitoring intervention groups [39,40]

### 3.4. mHealth

Of the 10 studies that had evaluated the effectiveness of mHealth interventions for type 2 diabetes management, 4 studies [5,31,41,42] reported statistically significant changes between the mHealth intervention groups and control groups measured by a reduction in mean HbA1c levels. 

For the studies that failed to see statistically significant intergroup differences in mean HbA1c, some statistically significant improvements were seen in secondary outcome measures including an improvement in health related quality of life measured by the Assessment of Quality of Life-8D (AQol)-8D [43], and improved diabetes related self-management behaviours measured by the Health Education Impact Questionnaire (HeiQ) [44].

### 3.5. Virtual Consultation

Of the three studies that had utilised video teleconferencing instead of usual care for type 2 diabetes management, one study [34] reported statistically significant changes between the intervention group and control group measured by a reduction in mean HbA1c levels. 

For the studies that failed to see statistically significant intergroup differences in mean HbA1c, some statistically significant improvements were seen in secondary outcome measures including improved blood pressure control measured by a reduction in systolic blood pressure for the video conferencing intervention group [28].

### 3.6. Telephone Communication 

Of the three studies that had utilised telephone communication instead of usual care for type 2 diabetes management, all studies [35,45,46] reported statistically significant changes between the intervention group and control group measured by a reduction in mean HbA1c levels. Odnoletkova et al. [45] also reported statistically significant improvements in secondary outcome measures including total cholesterol, BMI and body weight. A significant reduction in Body Mass Index (BMI) and diabetes self-management behaviours measured by the Diabetes Self-Management Questionnaire (DSMQ) was observed by von Storch et al. [35]. Mental health related quality of life assessed by the SF36 version 2 questionnaire was also significantly improved for the intervention group in the study conducted by Williams et al. [46].

### 3.7. Video Education

Of the single study carried out by Gupta et al. [29] utilising video education in the place of usual care, a significant reduction in mean HbA1c was observed in the intervention group as well as statistically significant improvements in secondary outcome measures of body weight, BMI, waist circumference and fasting plasma glucose.

### 3.8. Meta-Analysis Results

**Figure 2:**
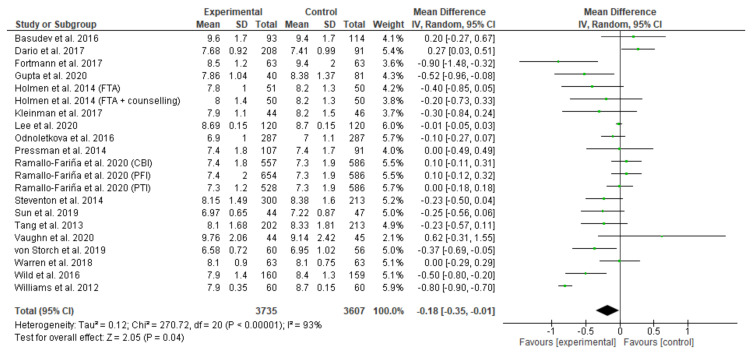
Mean HbA1c for all telehealth studies. BMI was used as a secondary outcome measure.

As outlined in Figure 2, a meta–analysis demonstrated that telehealth interventions significantly improved HbA1c levels compared to a control group, with a mean difference in HbA1c% −0.18 (CI −0.35, –0.01), *p* = 0.04. The heterogeneity between studies was very high (I^2^ = 93%). This finding is visually depicted on the forest plot below where the diamond sits to the left side of the plot in favour of telehealth interventions. As outlined in Appendix B, results from the subgroup meta-analysis for telemonitoring interventions found that telemonitoring led to improved HbA1c levels compared to a control group with a mean difference of −0.07 (CI −0.17, 0.03); however, it was not significant (*p* = 0.17), I^2^ = 60%. Similarly, subgroup analysis for mHealth interventions favoured mHealth over usual care with a mean difference of −0.04 (CI −0.14, 0.06); however it was not significant (*p* = 0.44) I^2^ = 59%. Subgroup analysis for the telephone communication interventions also favoured intervention over usual care with a mean difference of −0.43 (CI −0.94, 0.08); however, it was not significant (*p* = 0.10), I^2^ = 96%. Subgroup meta-analysis for the telehealth interventions that included self-management behaviours led to an improvement in HbA1c levels compared to usual care with a mean difference of −0.35 (CI −0.51, –0.18), *p* < 0.0001, I^2^ = 0%). 

Nine studies were included for the overall analysis which favoured usual care as opposed to telehealth interventions as a means to improve body mass index (BMI) amongst adults with type 2 diabetes. A random effects meta-analysis concluded that there was no statistical difference between the groups with a mean difference of 0.41 (CI −0.47, 1.29), *p* = 0.36, I^2^ = 65% (see Appendix B). Corresponding Forest plots for all the meta-analysis and subgroup analysis can be found in the Appendix (see Appendix B). 

## 4. Discussion 

Results from the meta-analysis indicate that telehealth interventions, irrespective of the telehealth technology used, had greater influence on reducing HbA1c levels compared to usual care. The narrative synthesis of the studies suggests that telemonitoring and telephone communication interventions had the greatest influence on HbA1c levels amongst adults living with type 2 diabetes. 

Whilst results from the meta-analysis favoured telehealth interventions over usual care for telemonitoring, telephone communication and mHealth interventions, none of these findings were statistically significant. As such, it is difficult to identify which of type of telehealth technology is most effective related to the management of type 2 diabetes. In a similar systematic review conducted by Lee et al. [47], the greatest effect on type 2 diabetes related outcomes was seen in telephone-delivered interventions, followed by Internet blood glucose monitoring system interventions, and lastly, interventions involving the automatic transmission of self-monitored blood glucose data (SMBG) using a mobile phone or a telehealth unit. 

Telemonitoring interventions included in this review varied in the type of technology used and the volume of health care provider input; however, interventions that demonstrated statistically significant changes in HbA1c incorporated some form of personalised feedback regarding type 2 diabetes management. This observation supports the findings from a systematic review conducted by Faruque et al. [48] who found that telemedicine interventions that facilitated more interaction between health care providers and patients were more likely to have greater effects on HbA1c levels. Our findings suggest that telemonitoring interventions incorporating personalised feedback into patient care are more likely to improve the effectiveness of the various types of telehealth interventions.

Whilst results from the subgroup meta-analysis for the mHealth interventions failed to demonstrate statistically significant differences in HbA1c, mHealth interventions had a greater effect on lowering HbA1c levels compared to usual care alone. From the selection of mHealth studies included in the systematic review, 4 of the 10 studies observed a significant reduction in mean HbA1c levels compared to controls. Whilst these studies varied in the design of mHealth interventions, all four studies shared the common feature of providing personalised feedback to participants.

Whilst age has been previously reported as a barrier towards telehealth engagement [49], it was not possible to further explore this relationship through meta-analysis as the available study data were not stratified by age group. 

Of the selected studies in the following systematic review, only 2 of the 29 studies reported age as an influential factor. Holmen et al. [44] reported that those over the age of 63 years demonstrated higher levels of engagement with the mobile app compared to their younger counter parts (*p* = 0.045). Sun et al. [5] reported that better treatment outcomes were observed in those over the age of 40 years for a combined telemonitoring and mHealth intervention. Of the selected studies in this review that demonstrated a significant reduction in HbA1c, the average age of participants was 59.1 years for telemonitoring interventions, 54.9 years for mHealth interventions, 59.8 years for telephone communication interventions, 58.1 years for virtual consultation and 50.2 years for the video education intervention. 

A recent systematic review by Tchero et al. [50] showed that patients aged 41–50 years or over 50 years were found to have more benefit from telemedicine interventions compared to younger age groups. The rationale behind these findings were not clear; however, they support the need for future telehealth studies to consider the effect of age within both the study design and the evaluative stages of research to better understand its effects. 

Of the 29 studies included in the systematic review, 9 studies had evaluated one or more components of type 2 diabetes self-management behaviour as a secondary outcome measure [8,30,31,35,37,39,44,51,52]. Results from the subgroup meta-analysis indicated that telehealth studies that utilised strategies to support the self-management behaviours of patients with type 2 diabetes saw significant improvement in HbA1c levels compared to the usual care groups (*p* < 0.0001). Whilst strategies to support the adoption and maintenance of self-management behaviours varied across the studies, these findings demonstrate that the inclusion of self-management behaviour strategies within telehealth interventions plays a critical role in lowering HbA1c levels. These strategies include regular interaction with a health care professional, personalised health coaching and goal setting around healthy lifestyle behaviours. 

Studies that observed significant improvements in HbA1c levels that had evaluated one or more components of self-management behaviours had utilised a form of telemonitoring for blood glucose levels as well as regular reporting for one or more forms of self-management behaviours. This real time sharing of blood glucose data and associated health information can create a sense of accountability for the patient as they may feel a greater responsibility to maintain daily blood glucose readings. This phenomenon can be explained by the Hawthorne effect (inclination to perform better when being watched) [53]. Whilst the Hawthorne effect is recognised as a form of bias in interventional studies, its potential application as a strategy to improve patient engagement regarding their diabetes management should not be overlooked [54]. 

Improvements in self-management related behaviours were reported across a number of studies in this review [8,31,35,44,52]. Change in self-management behaviours were assessed using tools such as the Diabetes Self-Management Questionnaire (DSMQ) [35] and the Diabetes Self Care Activities Scale [52]. In the mHealth study by [44], participants who had access to health counselling as a part of the mHealth intervention saw significant changes in self-management related behaviours compared to those who did not have access to health counselling. Improvements were also reported for specific diabetes self-management related domains such as medication adherence [31] and diet and foot care [8].

Whilst socioeconomic status (SES) as indicated by level of education or income were reported across a number of studies, the relationship between socioeconomic status and mean HbA1c was only evaluated in one of the selected studies [55], which found that telemonitoring combined with virtual case management improved HbA1c and systolic blood pressure outcomes in the lowest SES groups compared to higher SES groups. As it is well understood that type 2 diabetes disproportionately affects lower income communities [56], a number of the selected studies in this review have recruited subjects who were of a lower socioeconomic status or who belong to socially disadvantaged communities.

A recent study [57] found that median household income had a significant influence on telehealth engagement when virtual consultation was utilised in place of usual face-to-face care in primary health care settings, with those with lower median household incomes more likely to forgo an appointment. 

There were two studies in the included review that also considered the relationship between socioeconomic factors and their influence on telehealth efficacy; however, this relationship was not explicitly tested [4,41]. Both studies demonstrated significant improvements in HbA1c levels amongst a cohort of low-income earners for telemonitoring and mHealth telehealth interventions. As SES was not controlled for in these studies, it is not possible to examine the effect of socioeconomic factors on telehealth efficacy. This is an important finding as low SES has previously been linked to poorer health outcomes as a result of low formal education and low technical literacy which have been reported as barriers to telehealth engagement [49]. 

As patient and health care provider experiences have been shown to influence telehealth outcomes [58], we had intended to further explore this relationship in this review. Due to the inconsistencies in the assessment of perceived care and absence of associated data across studies, it was not possible to conduct a meta-analysis to further explore this relationship.

There were seven studies in this review that had evaluated perceived effectiveness of care or patient experiences of telehealth through participant surveys with questions focused around the use of telehealth technologies [5,8,31,35,40,41,42]. Whilst all these studies reported high levels of client satisfaction regarding telehealth intervention, only two studies formally tested treatment satisfaction between the intervention and control groups. Findings from Tang et al. [40] indicated that the intervention group had greater overall treatment satisfaction compared to the control group (*p* < 0.001), better knowledge regarding blood glucose testing (*p* < 0.004), and a better understanding of diabetes at 12 months (<0.001), whilst Kleinman et al. [31] reported no significant difference between the level of client satisfaction between intervention and control groups. 

## 5. Conclusions

This systematic review has supported previous findings regarding the effectiveness of telemonitoring interventions on improving the HbA1c levels in adults with type 2 diabetes, particularly where there are higher levels of health care provider involvement. This review also demonstrated that telehealth interventions that address type 2 diabetes self-management behaviours have a greater effect on lowering HbA1c levels compared to usual care alone. It is recommended that future randomised controlled trial studies in the area of telehealth and type 2 diabetes management examine the influence of age and socioeconomic status, consider longitudinal study designs to better understand long-term behaviour change effects and evaluate patient and health care provider experiences of telehealth to understand the feasibility of telehealth care models in primary care settings.

### Limitations

Not all potential randomised controlled trial studies were retrieved due to public access rights. It is important to note that whilst three telephone communication studies included in this review saw significant changes in mean HbA1c levels for adults living with type 2 diabetes, the small number of high-quality telephone-based intervention studies for type 2 diabetes management extracted makes it difficult to draw any strong conclusions. 

The search strategy utilised in this review may have excluded potential randomised control trial studies as we chose not to include comorbidity studies and studies that looked at type 1 or gestational diabetes. The decision to include such studies may have further strengthened the research findings. The search strategy in this review chose not to include specific types of telehealth technologies within the formal search, relying on the terms “telehealth” and “telemedicine” to capture all relevant articles. As such, it is recognized that potential studies may have been missed had they not been associated with these terms. This review also chose not to include the search terms “socioeconomic status” or “age effect” within the formal search strategy which have been reported as key barriers towards telehealth efficacy. This decision may have led to the lack of available data which was required to further explore the influence of socioeconomic status and age on telehealth efficacy.

## Figures and Tables

**Table 1:** Characteristics of the included RCT studies (*n* = 29).

Author/Year Country	Sample Size (*n*) Participant Characteristics (*% Male*)	Age (Years)	Description of Intervention	Length of Intervention	*p* value (95% CI) Mean HbA1c%
Argawal et al., 2019 Canada	*n* = 223 Intervention (110) *55%* (*1 participant gender not specified*) Control: (113) *49%*	Intervention 51.5 ± 10.6 Control 52.1 ± 10.7	**Telemonitoring + mHealth** Use of mobile app to enter data baseline health, blood glucose readings, exercise activity and food intake. App provided tailored messages to participants targeting motivation, education and behaviour change.	6 months	*p* = 0.19 CI (−1.05, 0.21)
Basudev et al., 2016 United Kingdom	*n* = 208 Intervention (93) *55%* Control (114) *60%*	Intervention 60.5 ± 12.3 Control 59.3 ± 12.0	**Virtual clinic** Virtual clinic model where participants have a virtual consultation with practice team for care planning related to type 2 diabetes management. Participants reviewed every 3 months.	12 months	*p* = 0.4 CI-not available
Dario et al., 2017 Italy	*n* = 299 Intervention (208) *57%* Control (91) *54%*	Intervention 73.05 ± 5.79 Control 73.04 ± 5.28	**Telemonitoring with feedback** Participants uploaded blood glucose readings to an online e-Health Centre. Clinicians monitor participant data through a Home Care portal with feedback provided to next of kin if indicated.	12 months	*p* = 0.76 CI (−0.2, 0.2)
Egede et al., 2017 USA	*n* = 113 Intervention (59) *17%* Control (54) *19%*	Intervention 55.1 ± 11.4 Control 53.4 ± 10.5	**Telemonitoring with feedback** Telemonitoring + case management via FORA 2 in 1 blood glucose and blood pressure monitoring system. Nurse case manager provides weekly medication adjustments based on daily participant data.	6 months	*p* = 0.024 CI (−1.86, −0.13)
Fortmann et al., 2017 USA	*n* = 126 Intervention (63) *27%* Control (63) *24%*	Intervention 47.8 ± 9.0 Control 49.1 ± 10.6	**mHealth** mHealth intervention involving text messages with motivational, educational and call to action messages 2–3 x day over a 6-month period. Participants encouraged to send blood glucose readings after text prompt.	6 months	*p* = 0.03 CI-not available
Gong et al., 2020 Australia	*n* = 187 Intervention (93) *53%* Control (94) *64%*	Intervention 55.4 ± 9.7 Control 58.4 ± 10.5	**mHealth** mHealth intervention involving mobile app which provides support, monitoring and motivational coaching via an embodied conversation agent.	12 months	*p* = 0.84 CI (−0.45, 0.36)
Greenwood et al., 2014 USA	*n* = 90 Intervention (45) *49%* Control (45) *58%*	Intervention 53.9 ± 10.4 Control 57.5 ± 10.6	**Telemonitoring with feedback** Telemonitoring with feedback which included a daily health session with an audible prompt for participant to take blood glucose readings with diabetes related education provided. Participants can access a diabetes clinical educator who is available via phone call or text to discuss diabetes care.	6 months	*p* < 0.001 CI-not available
Gupta et al., 2020 India	*n* = 81 Intervention (40) *45%* Control (41) 59%	Intervention 50.1 ± 9.4 Control 50.2 ± 8.6	**Video based education program** Video based lifestyle education program involving 12 modules over a 4-month period focused on type 2 diabetes health topics such as self-monitoring, diet, meal planning, exercise, etc.	4 months	*p* = 0.013 CI (0.14,1.14) *p* = 0.021 adjusted CI (0.10, 1.12)
Hansen et al., 2017 Denmark	*n* = 165 Intervention (83) *64%* Control (82) *65%*	Intervention 57.8 ± 9.4 Control 58.3 ± 9.3	**Video consultation + telemonitoring** Video consultation + telemonitoring involving 2 x monthly video conferences with a nurse via tablet computer for 32 weeks. Participants provided blood glucose readings, blood pressure readings and body weight readings to nurse.	8 months	*p* = 0.023 CI-not available
Holmen et al., 2014 Norway	*n* = 151 Intervention app (51) *67%* Intervention app + coaching (50) *50%* Control (50) *40%*	Intervention (app) 58.6 ± 11.8 Intervention (app + coaching) 57.4 ± 12.1 Control 55.9 ± 12.2	**mHealth** mHealth intervention with or without telephone counselling from diabetes specialist nurse for the first 4 months of the intervention. Mobile app was utilised for wireless transfer of blood glucose data, manual entry of diet, physical activity and personal goals related to type 2 diabetes management.	12 months	*p* = 0.42 (app) CI (−0.75, 0.32) *p* = 0.97(app + coaching) CI (–0.52, 0.54)
Kleinman et al., 2017 India	*n* = 91 Intervention (44) *82%* Control (46) *59%*	Intervention 48.8 ± 9.0 Control 48.0 ± 9.5	**mHealth** mHealth intervention involving mobile app reminding participants to complete blood glucose readings with access to a health coach. Out of range blood glucose levels prompted questions for participants and generated alerts to a health care team.	6 months	*p* = 0.02 CI (0.10, 1.37)
Lee et al., 2020 Malaysia	*n* = 240 Intervention (120) *44%* Control (120) *46%*	Intervention 56.1 ± 9.2 Control 56.3 ± 8.6	**Telemonitoring + case management** Telemonitoring + team-based management. Blood glucose levels uploaded to system, and care team adjusts medication accordingly. Healthy lifestyle education and advice also provided. Participants advised to report 6 glucose readings/week (3 prandial and 3 post-prandial). Feedback provided from care team if indicated.	12 months	*p* = 0.226 CI (−0.07, 0.02)
Lee et al., 2017 Malaysia	*n* = 85 Intervention (45) *53%* Control (40) 40%	Intervention 53.24 ± 7.29 Control 53.77 ± 8.03	**Telemonitoring + feedback** Blood glucose readings uploaded to an online portal via mobile device which is viewed by researcher. Reminders sent to participants to measure blood glucose levels. Case manager contacts participant to provide advice re: medication and lifestyle education.	3 months	*p* < 0.01 CI-not available
McLeod et al., 2020 New Zealand	*n* = 429 Intervention (215) *50%* Control (214) *48%*	Intervention 61.8 ± 9.5 Control 62.4 ± 8.7	**mHealth** mHealth intervention involving mobile device and web-based program with 4 components, health coaching, evidence-based resources, peer support and goal tracking. Health coaching for the first 4 months of intervention via text or fortnightly video meetings.	12 months	*p* = 0.990 CI (−0.1, 0.1)
Odnolekova et al., 2016 Belgium	*n* = 287 Intervention (287) *60%* Control (287) *63%*	Intervention 63.8 ±8.7 Control 62.4 ± 8.9	**Telephone communication “telecoaching”** Tele-coaching via telephone. Five nurse led telephone sessions averaging 30 mins every 3–8 weeks utilising motivational interviewing techniques.	6 months	*p* = 0.003 CI (−0.3, −0.1)
Parsons et al., 2019 United Kingdom	*n* = 323 Intervention (self-monitoring + feedback) (148) *59%* Intervention (self-monitoring) (147) *56%* Control (151) *58%*	Intervention (self-monitoring + telecare) 61.6 ±9.82 Intervention (self-monitoring) 62.9 ± 9.34 Control 60.7 ± 10.98	**Telemonitoring + feedback** Telemonitoring via diabetes management software, with or without telecare support via monthly phone call from diabetes nurse.	12 months	Intervention (SM + telecare) *p* < 0.0001 CI (−1.40, −0.94) Intervention (SM only) *p* < 0.0001 CI (–1.29, –0.81)
Pressman et al., 2014 USA	*n* = 225 Intervention (107) *63%* Control (91) *60%*	Intervention 54.8 ± 9.8 Control 56.4 ± 8.7	**Telemonitoring + feedback** Telemonitoring of blood glucose levels along with weekly communication with diabetes nurse.	6 months	*p* = 0.310 CI-not available
Ramallo-Fariña et al., 2020 Canary Islands	*n* = 2334 Intervention 1 PTI (587) *53%* Intervention 2 PFI (654) *56%* Intervention 3 CBI (557) *47%* Control (586) *49%*	Intervention (1) 55.9 ± 7.0 Intervention (2) 56.2 ± 7.0 Intervention (3) 55.5 ± 7.1 Control 55.2 ± 7.3	**Telemonitoring via web platform with SMS (mHealth)** Telemonitoring via web-based platform with automated SMS. Participants required to log blood glucose levels for feedback. Intervention also involved health care provider training regarding telehealth intervention.	24 months	*p* = 0.3 (PTI) CI (−0.48, −0.04) *p* = 0.9 (PFI) CI (−0.41, 0.03) *p* = 0.3 (CBI) CI (−0.47, −0.03)
Shea et al., 2013	Data obtained from Weinstock et al., 2011				
Steventon et al., 2014 United Kingdom	*n* = 513 Intervention (300) *53%* Control (213) *64%*	Intervention 63.9 ± 13.0 Control 66.2 ± 11.9	**Telemonitoring + automated SMS feedback** Telemonitoring with feedback when indicated. Participants encouraged to upload blood glucose data with automated educational messages provided.	12 months	*p* = 0.125 unadjusted CI (−0.60, 0.07) *p* = 0.0009 adjusted CI (−0.52, −0.07)
Sun et al., 2019 China	*n* = 91 Intervention (44) *43%* Control (47) *38%*	Intervention 67.9 ± 2.5 Control 68.04 ± 3.0	**Telemonitoring + mHealth** Telemonitoring combined with mHealth. Daily wireless transmission of blood glucose data through glucometer along with feedback via telemedicine system regarding management of type 2 diabetes.	6 months	*p* = 0.02 CI-not available
Tang et al., 2013 USA	*n* = 415 Intervention (202) *59%* Control (213) *61%*	Intervention 54.0 ± 10.7 Control 53.5 ± 10.2	**Telemonitoring + feedback** Telemonitoring + personalised feedback as well as personalised text and video educational content.	12 months	*p* = 0.133 CI-not available
Trief et al., 2013	Data obtained from Weinstock et al., 2011				
Vaughan et al., 2020 USA	*n* = 89 Intervention (44) *23%* Control (45) 33%	Intervention 55.99 ± 7.12 Control 53.86 ± 9.07	**mHealth** mHealth intervention, regular communication call or SMS with health care practitioner, diabetes group visits with community health worker.	6 months	unadjusted *p* = 0.007 CI-not available Adjusted *p* = 0.002 CI-not available
Von Storch et al., 2019 Germany	*n* = 115 Intervention (60) *78%* Control (56) *84%*	Intervention 59.4 ± 6.3 Control 58.4 ± 7.3	**Telemonitoring + telephone communication** Telemonitoring of blood glucose levels along with feedback if indicated utilising tablet computer, glucometer and step counter with monthly telephone coaching.	12 months (results reported at 3 months)	*p* = 0.038 CI-not available
Warren et al., 2018 Australia	*n* = 126 Intervention (63) *60%* Control (63) *48%*	Intervention 61.3 ± 10.8 Control 61.3 ± 11.4	**Telemonitoring + feedback** Telemonitoring of blood glucose levels along with feedback.	12 months	*p* = 0.004 CI-not available
Weinstock et al., 2011 USA	*n* = 1665 Intervention (844) 36% Control (821) 38%	Intervention 70.8 ± 6.5 Control 70.9 ± 6.8	**Telemonitoring + video conferencing** Telemonitoring of blood glucose levels + video consultation with health care provider.	5 years	*p* = 0.001 CI-not available
Wild et al., 2016 United Kingdom	*n* = 321 Intervention (160) *66%* Control (159) 67%	Intervention 60.5 ± 9.8 Control 61.4± 9.8	**Telemonitoring + feedback** Telemonitoring of blood glucose le levels + once weekly feedback/review with diabetes educator.	9 months	*p* = 0.007 CI (0.22, 0.81)
Williams et al., 2012 Australia	*n* = 120 Intervention (60) *62%* Control (60) *63%*	Intervention 58.4 ± 8.2 Control 56.4 ± 8.3	**Telephone communication** Automated telephone management intervention, with 1 × weekly phone calls lasting 5–20 minutes and education provided on a variety of diabetes related topics.	6 months	*p* = 0.002 CI (0.86–0.93)

## Data Availability

Data is contained within the article.

## Appendix A. Cochrane Back Review Group Risk of Bias Assessmen

Risk of Bias Assessment using the Cochrane Back Review Group Assessment Criteria	Argawal et al., 2019	Basudev et al., 2016	Dario et al., 2017	Egede et al., 2017	Fortmann et al., 2017	Gong et al., 2020	Greenwood et al., 2014	Gupta et al., 2020	Hansen et al., 2017	Holmen et al., 2014	Kleinman et al., 2017	Lee et al., 2020	Lee et al., 2017	Mcleod et al., 2020	Odnoletkova et al., 2016	Parsons et al., 2019	Pressman et al., 2014	Ramallo-Fariña et al., 2020	Shea et al., 2013	Steventon et al., 2014	Sun et al., 2019	Tang et al., 2013	Trief et al., 2013	Vaughn et al., 2020	von Storch et al., 2019	Warren et al., 2018	Weinstock et al., 2011	Wild et al., 2016	Williams et al., 2012
1. Was the method of randomisation adequate?																													
2. Was the treatment allocation concealed?																													
3. Was the patient blinded to the intervention?																													
4. Was the care provider blinded to the intervention?																													
5. Was the outcome assessor blinded to the intervention?																													
6. Was the dropout rate described and acceptable?																													
7. Were all randomised participants analysed in the group to which they were allocated?																													
8. Are reports of the study free of suggestion of selective outcome reporting?																													
9. Were the groups similar at baseline regarding the most important prognostic indicators?																													
10. Were co-interventions avoided or similar?																													
11. Was the compliance acceptable in all groups?																													
12. Was the timing of the outcome assessment similar in all groups?																													
Key:	Yes									No										Unsure									

## References

[1] International Organisation for Standardization (2021). Health Informatics—Capacity-Based Ehealth Architecture Roadmap—Part 1: Overview of National Ehealth Initiatives.

[2] World Health Organisation (2009). Telemedicine: Opportunities and Developments in Member States, in Global Observatory for Ehealth Series.

[3] Dinesen B., Nonnecke B., Lindeman D., Toft E., Kidholm K., Jethwani K., Young H.M., Spindler H., Oestergaard C.U., Southard J.A. (2016). Personalized telehealth in the future: A global research agenda. J. Med. Internet Res..

[4] Egede L.E., Williams J.S., Voronca D.C., Knapp R.G., Fernandes J.K. (2017). Randomized controlled trial of technology-assisted case management in low income adults with type 2 diabetes. Diabetes Technol. Ther..

[5] Sun C., Sun L., Xi S., Zhang H., Wang H., Feng Y., Deng Y., Wang H., Xiao X., Wang G. (2019). Mobile phone-based telemedicine practice in older chinese patients with type 2 diabetes mellitus: Randomized controlled trial. JMIR Mhealth Uhealth.

[6] Ansari R.M., Hosseinzadeh H., Harris M., Zwar N. (2019). Self-management experiences among middle-aged population of rural area of Pakistan with type 2 diabetes: A qualitative analysis. Clin. Epidemiol. Glob. Health.

[7] Yadav U.N., Lloyd J., Hosseinzadeh H., Baral K.P., Dahal S., Bhatta N., Harris M.F. (2020). Facilitators and barriers to the self-management of COPD: A qualitative study from rural Nepal. BMJ Open.

[8] Greenwood D.A. (2014). Evaluation of a Telehealth Intervention Combining Structured Self-Monitoring of Blood Glucose and Nurse Care Coordination among People with Type 2 Diabetes Noninsulin-Treated.

[9] Weinstock R.S., Teresi J.A., Goland R., Izquierdo R., Palmas W., Eimicke J.P., Ebner S., Shea S. (2011). Glycemic control and health disparities in older ethnically diverse underserved adults with diabetes: Five-year results from the Informatics for Diabetes Education and Telemedicine (IDEATel) study. Diabetes Care.

[10] Bali S. (2019). Barriers to Development of Telemedicine in Developing Countries.

[11] Smith A.C., Snoswell C.L., Mehrotra A., Thomas E., Haydon H., Clemensen J., Caffery L.J. (2020). Telehealth for global emergencies: Implications for coronavirus disease 2019 (COVID-19). J. Telemed. Telecare.

[12] Su D., Zhou J., Kelley M.S., Michaud T.L., Siahpush M., Kim J., Wilson F., Stimpson J.P., Paga´n J.A. (2016). Does telemedicine improve treatment outcomes for diabetes? A meta-analysis of results from 55 randomized controlled trials. Diabetes Res. Clin. Pract..

[13] International Diabetes Federation (2019). IDF Diabetes Atlas.

[14] WHO Reveals Leading Cause of Death and Disability Worldwide 2000–2019. https://www.who.int/news/item/09-12-2020-who-reveals-leading-causes-of-death-and-disability-worldwide-2000-2019.

[15] Royal Australian College of General Practitioners (2020). Management of Type 2 Diabetes: A Handbook for General Practice.

[16] Almutairi N., Hosseinzadeh H., Gopaldasani V. (2020). The effectiveness of patient activation intervention on type 2 diabetes mellitus glycemic control and self-management behaviors: A systematic review of RCTs. Prim. Care Diabetes.

[17] Ansari R.M., Harris M., Hosseinzadeh H., Zwar N. (2021). Healthcare professionals’ perspectives of patients’ experiences of the self-management of type 2 diabetes in the rural areas of pakistan: A qualitative analysis. Int. J. Environ. Res. Public Health.

[18] Hosseinzadeh H., Verma I., Gopaldasani V. (2020). Patient activation and type 2 diabetes mellitus self-management: A systematic review and meta-analysis. Aust. J. Prim. Health.

[19] Edwards J., Hosseinzadeh H. (2018). The impact of structured physical activity on glycaemic control in diabetes prevention programmes: A systematic review. Proc. Singap. Healthc..

[20] Hosseinzadeh H., Shnaigat M. (2019). Effectiveness of chronic obstructive pulmonary disease self-management interventions in primary care settings: A systematic review. Aust. J. Prim. Health.

[21] Niknami M., Mirbalouchzehi A., Zareban I., Kalkalinia E., Rikhtgarha G., Hosseinzadeh H. (2018). Association of health literacy with type 2 diabetes mellitus self-management and clinical outcomes within the primary care setting of Iran. Aust. J. Prim. Health.

[22] Esmatjes E., Jansà M., Roca D., Pérez-Ferre N., Del Valle L., Martínez-Hervás S., Ruiz De Adana M., Linares F., Batanero R., Vázquez F. (2014). The efficiency of telemedicine to optimize metabolic control in patients with type 1 diabetes mellitus: Telemed study. Diabetes Technol..

[23] González-Molero I., Domínguez-López M., Guerrero M., Carreira M., Caballero F.F., Rubio-Martín E., Linares F., Cardona I., Anarte Ortiz M.T., Adana M. (2012). Use of telemedicine in subjects with type 1 diabetes equipped with an insulin pump and real-time continuous glucose monitoring. J. Telemed. Telecare.

[24] Landau Z., Mazor-Aronovitch K., Boaz M., Blaychfeld-Magnazi M., Graph-Barel C., Levek-Motola N., Pinhas-Hamiel O. (2012). The effectiveness of Internet-based blood glucose monitoring system on improving diabetes control in adolescents with type 1 diabetes. Pediatric Diabetes.

[25] Furlan A.D., Pennick V., Bombardier C., van Tulder M. (2009). 2009 updated method guidelines for systematic reviews in the cochrane back review group. SPINE.

[26] Corbin L.J., Richmond R.C., Wade K.H., Burgess S., Bowden J., Davey Smith G., Timpson N.J. (2016). BMI as a modifiable risk factor for type 2 diabetes: Refining and understanding causal estimates using mendelian randomization. Diabetes.

[27] Ryan R. (2016). Cochrane Consumers and Communication Group reviews: Meta-analysis. Cochrane Handbook for Systematic Reviews of Interventions.

[28] Basudev N., Crosby-Nwaobi R., Thomas S., Chamley M., Murrells T., Forbes A. (2016). A prospective randomized controlled study of a virtual clinic integrating primary and specialist care for patients with type 2 diabetes mellitus. Diabetes Med..

[29] Gupta U., Gupta Y., Jose D., Mani K., Jyotsna V.P., Sharma G., Tandon N. (2020). Effectiveness of a video-based lifestyle education program compared to usual care in improving hba1c and other metabolic parameters in individuals with type 2 diabetes: An open-label parallel arm randomized control trial (RCT). Diabetes Ther..

[30] Agarwal P., Mukerji G., Desveaux L., Ivers N.M., Bhattacharyya O., Hensel J.M., Shaw J., Bouck Z., Jamieson T., Onabajo N. (2019). Mobile app for improved self-management of type 2 diabetes: Multicenter pragmatic randomized controlled trial. JMIR Mhealth Uhealth.

[31] Kleinman N.J., Shah A., Shah S., Phatak S., Viswanathan V. (2017). Improved medication adherence and frequency of blood glucose self-testing using an m-health platform versus usual care in a multisite randomized clinical trial among people with type 2 diabetes in India. Telemed. J. E Health.

[32] Ramallo-Fariña Y., García-Bello M.A., García-Pérez L., Boronat M., Wägner A.M., Rodríguez-Rodríguez L., de Pablos-Velasco P., Llorente Gómez de Segura I., González- Pacheco H., Carmona Rodríguez M. (2020). Effectiveness of internet-based multicomponent interventions for patients and health care professionals to improve clinical outcomes in type 2 diabetes evaluated through the INDICA study: Multiarm cluster randomized controlled trial. JMIR Mhealth Uhealth.

[33] Steventon A., Bardsley M., Doll H., Tuckey E., Newman S.P. (2014). Effect of telehealth on glycaemic control: Analysis of patients with type 2 diabetes in the Whole Systems Demonstrator cluster randomised trial. BMC Health Serv. Res..

[34] Hansen C.R., Perrild H., Koefoed B.G., Zander M. (2017). Video consultations as add-on to standard care among patients with type 2 diabetes not responding to standard regimens: A randomized controlled trial. Eur. J. Endocrinol..

[35] Von Storch K., Graaf E., Wunderlich M., Rietz C., Polidori M.C., Woopen C. (2019). Telemedicine-assisted self-management program for type 2 diabetes patients. Diabetes Technol. Ther..

[36] Parsons S.N., Luzio S.D., Harvey J.N., Bain S.C., Cheung W.Y., Watkins A., Owens D.R. (2019). Effect of structured self-monitoring of blood glucose, with and without additional TeleCare support, on overall glycaemic control in non-insulin treated Type 2 diabetes: The SMBG Study, a 12-month randomized controlled trial. Diabetes Med..

[37] Wild S.H., Hanley J., Lewis S.C., McKnight J.A., McCloughan L.B., Padfield P.L., Parker R.A., Paterson M., Pinnock H., Sheikh A. (2016). Supported telemonitoring and glycemic control in people with type 2 diabetes: The telescot diabetes pragmatic multicenter randomized controlled trial. PLoS Med..

[38] Dario C., Toffanin R., Calcaterra F., Saccavini C., Stafylas P., Mancin S., Vio E. (2017). Telemonitoring of type 2 diabetes mellitus in Italy. Telemed. J. e-Health.

[39] Pressman A.R., Kinoshita L., Kirk S., Barbosa G.M., Chou C., Minkoff J. (2014). A novel telemonitoring device for improving diabetes control: Protocol and results from a randomized clinical trial. Telemed. J. e-Health.

[40] Tang P.C., Overhage M.J., Chan A.S., Brown N.L., Aghighi B., Entwistle M.P., Hui S.L., Hyde S.M., Klieman L.H., Mitchell C.J. (2013). Online disease management of diabetes: Engaging and motivating patients online with enhanced resources-diabetes (EMPOWER-D), a randomized controlled trial. J. Am. Med. Inform. Assoc..

[41] Fortmann A.L., Gallo L.C., Garcia M.I., Taleb M., Euyoque J.A., Clark T., Skidmore J., Ruiz M., Dharkar-Surber S., Schultz J. (2017). Dulce digital: An mhealth sms-based intervention improves glycemic control in hispanics with type 2 diabetes. Diabetes care.

[42] Vaughan E.M., Hyman D.J., Naik A.D., Samson S.L., Razjouyan J., Foreyt J.P. (2020). A telehealth-supported, integrated care with CHWs, and MEdication-access (TIME) program for diabetes improves HbA1c: A randomized clinical trial. J. Gen. Intern. Med..

[43] Gong E., Baptista S., Russell A., Scuffham P., Riddell M., Speight J., Bird D., Williams E., Lotfaliany M., Oldenburg B. (2020). My diabetes coach, a mobile app based interactive conversational agent to support type 2 diabetes self-management: Randomized effectiveness-implementation trial. J. Med. Internet Res..

[44] Holmen H., Torbjørnsen A., Wahl A.K., Jenum A.K., Småstuen M.C., Årsand E., Ribu L. (2014). A mobile health intervention for self-management and lifestyle change for persons with type 2 diabetes, part 2: One-year results from the norwegian randomized controlled trial RENEWING HEALTH. JMIR mHealth uHealth.

[45] Odnoletkova I., Goderis G., Nobels F., Fieuws S., Aertgeerts B., Annemans L., Ramaekers D. (2016). Optimizing diabetes control in people with Type 2 diabetes through nurse-led telecoaching. Diabetes Med..

[46] Williams E.D., Bird D., Forbes A.W., Russell A., Ash S., Friedman R., Scuffham P.A., Oldenburg B. (2012). Randomised controlled trial of an automated, interactive telephone intervention (TLC Diabetes) to improve type 2 diabetes management: Baseline findings and six-month outcomes. BMC Public Health.

[47] Lee P.A., Greenfield G., Pappas Y. (2018). The impact of telehealth remote patient monitoring on glycemic control in type 2 diabetes: A systematic review and meta-analysis of systematic reviews of randomised controlled trials. BMC Health Serv. Res..

[48] Faruque L.I., Wiebe N., Ehteshami-Afshar A., Liu Y., Dianati-Maleki N., Hemmelgarn B.R., Manns B.J., Tonelli M. (2017). Effect of telemedicine on glycated hemoglobin in diabetes: A systematic review and meta-analysis of randomized trials. CMAJ.

[49] Alvarado M.M., Kum H.C., Gonzalez Coronado K., Foster M.J., Ortega P., Lawley M.A. (2017). Barriers to remote health interventions for type 2 diabetes: A systematic review and proposed classification scheme. J. Med. Internet Res..

[50] Tchero H., Kangambega P., Briatte C., Brunet-Houdard S., Retali G.R., Rusch E. (2019). Clinical effectiveness of telemedicine in diabetes mellitus: A meta-analysis of 42 randomized controlled trials. Telemed. e-Health.

[51] McLeod M.S., Signal J., Stairmand V., Thompson J., Henderson D., Davies K., Krebs C., Dowell J., Grainger A.R. (2020). Impact of a comprehensive digital health programme on HbA1c and weight after 12 months for people with diabetes and prediabetes: A randomised controlled trial. Diabetology.

[52] Trief P.M., Izquierdo R., Eimicke J.P., Teresi J.A., Goland R., Palmas W., Shea S., Weinstock R.S. (2013). Adherence to diabetes self care for white, African-American and Hispanic American telemedicine participants: 5 year results from the IDEATel project. Ethn. Health.

[53] McCambridge J., Witton J., Elbourne D.R. (2014). Systematic review of the Hawthorne effect: New concepts are needed to study research participation effects. J. Clin. Epidemiol..

[54] Oussedik E., Foy C.G., Masicampo E.J., Kammrath L.K., Anderson R.E., Feldman S.R. (2017). Accountability: A missing construct in models of adherence behavior and in clinical practice. Patient Prefer. Adherence.

[55] Shea S., Kothari D., Teresi J.A., Kong J., Eimicke J.P., Lantigua R.A., Palmas W., Weinstock R.S. (2013). Social impact analysis of the effects of a telemedicine intervention to improve diabetes outcomes in an ethnically diverse, medically underserved population: Findings from the IDEATel Study. Am. J. Public Health.

[56] Stringhini S., Batty G.D., Bovet P., Shipley M.J., Marmot M.G., Kumari M., Tabak A.G., Kivimaki M. (2013). Association of lifecourse socioeconomic status with chronic inflammation and type 2 diabetes risk: The Whitehall II prospective cohort study. PLoS Med..

[57] Darrat I., Tam S., Boulis M., Williams A.M. (2021). Socioeconomic disparities in patient use of telehealth during the coronavirus disease 2019 surge. JAMA Otolaryngol. Head Neck Surg..

[58] Radhakrishnan K., Xie B., Berkley A., Kim M. (2016). Barriers and facilitators for sustainability of tele-homecare programs: A systematic review. Health Serv. Res..

